# Temporal trends in prevalence and outcomes of atrial fibrillation in patients undergoing percutaneous coronary intervention

**DOI:** 10.1002/clc.23285

**Published:** 2019-11-06

**Authors:** Yusuke Morita, Toka Hamaguchi, Yuhei Yamaji, Hideyuki Hayashi, Eisaku Nakane, Yoshisumi Haruna, Tetsuya Haruna, Michiya Hanyu, Moriaki Inoko

**Affiliations:** ^1^ Cardiovascular Center, Tazuke Kofukai Foundation Medical Research Institute, Kitano Hospital Osaka Japan

**Keywords:** atrial fibrillation, percutaneous coronary intervention, epidemiology

## Abstract

**Background:**

Atrial fibrillation (AF) is the most common arrhythmia in patients undergoing percutaneous coronary intervention (PCI).

**Hypothesis:**

Large administrative data may provide further insight into temporal trends in the prevalence and burden of AF in patients who underwent PCI.

**Methods:**

Using the National Inpatient Sample database in the U.S., AF patients ≥18 years who underwent PCI between 2005 and 2014 and were identified by the International Classification of Diseases, ninth revision, Clinical Modification, were examined. In‐hospital mortality, morbidity, resource use, and medical costs were evaluated in crude and propensity‐matched analyses.

**Results:**

Among an estimated 6 272 232 hospitalizations, of patients undergoing PCI, AF prevalence was 9.9% and steadily increased from 8.6% to 12.0% between 2005 and 2014 (*P* < .001); there was also a greater proportion of comorbidities. There was a marked increase in AF prevalence among those aged ≥65 years and those undergoing elective PCIs. AF was independently associated with higher in‐hospital mortality and higher rates of transient ischaemic attack/stroke, bleeding complications, and non‐home discharge. Excessive in‐hospital mortality, stroke rate, gastrointestinal bleeding, blood transfusion, length of stay, and costs among AF hospitalizations were consistently observed throughout the study period.

**Conclusion:**

AF becomes more prevalent in patients undergoing PCI, possibly due to a higher comorbidity, particularly in elderly patients with non‐acute indications. Less favorable trends in mortality, bleeding, and stroke among AF patients who underwent PCI were consistent over time. Continuous efforts are needed to improve outcomes and manage strategies for AF patients undergoing PCI.

**Abbreviations list**
AF: atrial fibrillation
ICD‐9‐CM: international classification of diseases, ninth edition, clinical modification
STEMI: ST‐segment elevation myocardial infarction
NSTEMI: non‐ST‐segment elevation myocardial infarction
NIS: national inpatient Sample
PCI: percutaneous coronary intervention
TIA: transient ischemic attack

## 1 INTRODUCTION

Atrial fibrillation (AF) is the most common type of arrhythmia. The prevalence of AF in the general population of the US is 1% among all adults and 9% for those older than 80 years.^1^ Approximately 5%‐10% of patients who have undergone percutaneous coronary intervention (PCI) or acute coronary syndrome have concomitant AF.^2^, ^3^, ^4^, ^5^ These patients frequently require anticoagulants plus dual antiplatelet therapy and typically have significant in‐hospital morbidity and mortality.^2^, ^6^

Until recently, considerable efforts have been made to determine the optimal antithrombotic therapeutic approach for simultaneously preventing thromboembolic events and bleeding complications.^7^, ^8^, ^9^, ^10^, ^11^, ^12^, ^13^ The implementation of PCIs has changed in recent years, with second‐generation drug‐eluting stents being widely used and patients with greater comorbidity burdens undergoing procedures.^14^, ^15^ As the prevalence of AF in the US is expected to double from 2010 to 2050 due to an aging society,^1^ the management of AF patients undergoing PCI will impose a large healthcare burden. However, there are minimal data based on nationally representative cohorts in the US that focus on contemporary trends in the prevalence, in‐hospital outcomes, and medical costs of AF patients who have undergone PCI.

The aims of this study were to describe (a) changing trends in the prevalence of AF among patients who underwent PCI, (b) patient/hospital characteristics and clinical risk profiles of these patients, and (c) temporal trends and outcomes of in‐hospital mortality, in‐hospital morbidity, medical costs, and resource use among propensity‐matched cohorts of AF and non‐AF patients who underwent PCI.

## 2 METHODS

### 2.1 Study data

We used the National Inpatient Sample (NIS) database derived from administrative data between 2005 and 2014 for this retrospective, observational study. The NIS is the largest publicly available, all‐payer administrative database in the US and contains information regarding patient discharge from around 4500 hospitals in ~45 states.^16^ This database contains clinical and resource use information on roughly 7 million unweighted discharges annually, which represents around 35 million weighted discharges for national estimates. We calculated the national estimates using the discharge weights provided in the NIS. The NIS represents ~20% of stratified US in‐patient hospitalizations and excludes rehabilitation and long‐term care hospitals. A variety of studies for the association of clinical settings and procedures can be conducted using the NIS.^17^, ^18^ Because ICD code 9CM was changed to ICD10 in NIS after the middle of 2015, we used data from the 10 years leading up to 2014, prior to the ICD coding change. Institutional review board approval and informed consent were waived because the NIS is a publicly available database that contains completely de‐identified patient information.

### 2.2 Study population

We used international classification of diseases, ninth edition, clinical modification (ICD‐9‐CM) procedure codes (0066, 3601, 3602, 3605, 3606, 3607, 1755) to identify all hospitalizations of ages ≥18 years who underwent PCI (weighted n = 6 515 521; unweighted n = 1 322 243). The validation and use of the ICD‐9‐CM codes for identifying PCI numbers in the US has been described in previous studies.^19^, ^20^ After excluding hospitalizations with missing values for in‐hospital death, length of stay, discharge location, primary insurance, and in‐hospital costs, the final cohort included 6 272 232 hospitalizations (unweighted n = 1 272 853). As the variable for race was missing in 18% of hospitalizations, we performed multiple imputation using the R package mi (version 1.0),^21^ which imputes missing values in an approximate Bayesian framework. Hospital bed size, location, region, and median household income were entered into the model. The study cohort was divided into two groups according to the presence or absence of AF using ICD‐9‐CM code 42731, which was used in similar studies.^22^, ^23^ Elixhauser Comorbidity Software (version 3.7) was used to identify congestive heart failure, peripheral artery disease, diabetes, chronic pulmonary disease, chronic renal failure, obesity, anemia, and depression.^24^ Clinical Classifications Software for the ICD‐9‐CM was used to identify dyslipidemia and blood transfusion.^25^ Other ICD‐9‐CM codes for identifying patient/hospital characteristics are summarized in Table S1.

### 2.3 Propensity score matching

To reduce any bias associated with patient/hospital characteristics among AF and non‐AF hospitalizations, we performed 1:1 the nearest‐neighbor matching with a caliper of 0.15 using the MatchIt R package (v3.0.2).^26^ A total of 27 covariates of baseline characteristics were used for propensity matching, including age, sex, weekend admission, indications for PCI, primary insurance, median household income, location/teaching status of hospitals, bed size, region, family history of coronary artery disease, prior myocardial infarction, prior PCI, prior coronary artery bypass grafting, carotid artery disease, smoking history, hypertension, congestive heart failure, peripheral artery disease, diabetes, chronic pulmonary disease, chronic renal failure, obesity, anemia, dyslipidemia, depression, and dementia (Table 1 and Figure S1). Absolute standardized differences of ≤10% indicated relatively small imbalances in baseline characteristics.^27^

**Table 1:** Baseline patient and hospital characteristics for hospitalizations with and without atrial fibrillation undergoing percutaneous coronary intervention

	Crude			Propensity‐matched		
Characteristic	No AF	AF	*P* value	No AF	AF	*P* value
N, Unweighted	1 147 084	125 769		125 769	125 769	
Age in years (IQR)	64 (55‐73)	74 (66‐81)	<.001	74 (66‐81)	74 (66‐81)	.5
Women (%)	33.5	35.4	<.001	35.4	35.4	.91
Weekend admission (%)	16.3	17.6	<.001	17.4	17.6	.24
Indication (%)			<.001			.99
STEMI	22.7	21		21	21	
NSTEMI	44.1	44		44	44	
Elective	33.2	35		35	35	
Race (%)			<.001			.45
White	78.8	85.5		85.3	85.5	
Black	8.3	5.1		5.1	5.1	
Hispanic	6.7	4.7		4.8	4.7	
Asian or Pacific Islander	2.1	1.6		1.7	1.6	
Native American	0.5	0.4		0.4	0.4	
Other	3.6	2.7		2.7	2.7	
Primary insurance (%)			<.001			.02
Medicare	48.9	74.4		75	74.4	
Medicaid	6.2	3		3	3	
Private insurance	36.1	18.6		18.1	18.6	
Self‐pay	5.4	2.1		2	2.1	
Other	3.6	2.7		2.7	2.7	
No charge	0.5	0.2		0.2	0.2	
Median household income, percentile (%)			<.001			.91
0‐25th	27.1	25.4		25.5	25.4	
26th‐50th	26.8	27.4		27.4	27.4	
51st‐75th	24.6	25.2		25.1	25.2	
76th‐100th	21.4	22		21.9	22	
Location and teaching status (%)			<.001			.85
Rural	5.5		<.001	5.8	5.7	
Urban, non‐teaching	40.2		<.001	40.9	40.8	
Urban, teaching	54.3		<.001	53.4	53.5	
Bed size (%)			.007			.7
Small	8	8.3		8.2	8.3	
Medium	20.8	20.9		21	20.9	
Large	71.2	70.8		70.8	70.8	
Region (%)			<.001			.49
Northeast	18.3	17.8		17.6	17.8	
Midwest	24.9	26		26.1	26	
South	40.2	39.4		39.4	39.4	
West	16.7	16.8		16.9	16.8	
Comorbidities (%)						
Family history of CAD	10	4.8	<.001	4.6	4.8	.01
Prior MI	12.8	11.9	<.001	11.9	11.9	.86
Prior PCI	18.6	15.3	<.001	15.4	15.3	.43
Prior CABG	6.9	7.8	<.001	8	7.8	.11
Carotid artery disease	1.7	2.2	<.001	2.2	2.2	.49
Smoking history	35.9	22.6	<.001	22.6	22.6	.72
Hypertension	71.5	72.1	<.001	72.4	72.1	.1
Congestive heart failure	13.2	33.4	<.001	32.1	33.4	<.001
Peripheral artery disease	9.8	12.5	<.001	12.6	12.5	.31
Diabetes	33.1	32.8	.03	33.1	32.8	.21
Chronic pulmonary disease	14.7	20.9	<.001	20.8	20.9	.78
Chronic renal failure	9.2	16.3	<.001	16.1	16.3	.1
Obesity	12.2	10.5	<.001	10.5	10.5	.78
Anemia	7.1	11.2	<.001	11	11.2	.14
Dyslipidemia	67	57.2	<.001	57.8	57.2	.003
Depression	5.3	4.4	<.001	4.5	4.4	.96
Dementia	1.2	2.6	<.001	2.6	2.6	.18

Abbreviations: AF, atrial fibrillation; CABG, coronary artery bypass grafting; CAD, coronary artery disease; CI, confidence interval; IQR, interquartile range; MI, myocardial infarction; NSTEMI, non‐STEMI; OR, odds ratio; PCI, percutaneous coronary intervention; STEMI, ST‐segment elevation myocardial infarction; TIA, transient ischemic attack.

### 2.4 Primary outcome measures

The primary outcomes analyzed were (a) in‐hospital mortality; (b) in‐hospital morbidity (transient ischemic attack [TIA]/stroke, gastrointestinal bleeding, vascular complications, blood transfusion, cardiogenic shock, deep venous thrombosis, pulmonary embolism, and acute kidney injury); (c) length‐of‐stay; (d) non‐home discharge; and (e) in‐hospital cost. These outcomes were compared between crude and propensity‐matched cohorts, both with and without AF.

### 2.5 Statistical analysis

In‐hospital outcome and trend analyses were performed in unweighted and weighted data, respectively, which are a standard methodology used in other studies using NIS data.^23^, ^28^ The total charges provided in the NIS of each hospitalization were converted to cost estimates using the group average, all‐payer, in‐patient, cost‐to‐charge ratios. All in‐hospital costs were converted to projected estimates for the year 2014 using annual inflation rates on the basis of consumer price index data available from the Bureau of Labor Statistics.^29^

For trend analysis, the Mann‐Kendall test was performed for proportions and continuous data during the study period. Categorical variables are presented as percentages. Continuous variables are presented as mean ± SD or median (interquartile range) according to the normality of distribution. The Shapiro‐Wilk test was used to examine the normality of distribution. Patient/hospital characteristics and in‐hospital outcomes were compared using the Student's *t* test, Mann‐Whitney *U*‐test, and chi‐square test for normally distributed continuous variables, non‐normally distributed continuous variables, and categorical variables, respectively. In‐hospital outcomes for propensity‐matched cohorts were assessed by multivariate logistic regression analysis using variables included for propensity matching. To detect the significant timing of the changing slope in AF prevalence during the study period, we used Davies test (k = 10) by segmented R package (v0.5.3.0).^30^ *P* values <.05 were considered statistically significant, and all statistical analyses were two‐sided and performed using R software (version 3.4.1, R Foundation for Statistical Computing, Vienna, Austria). The R package survey (version 3.33) was used to analyze the weighted database.^31^

## 3 RESULTS

**Figure 1:**
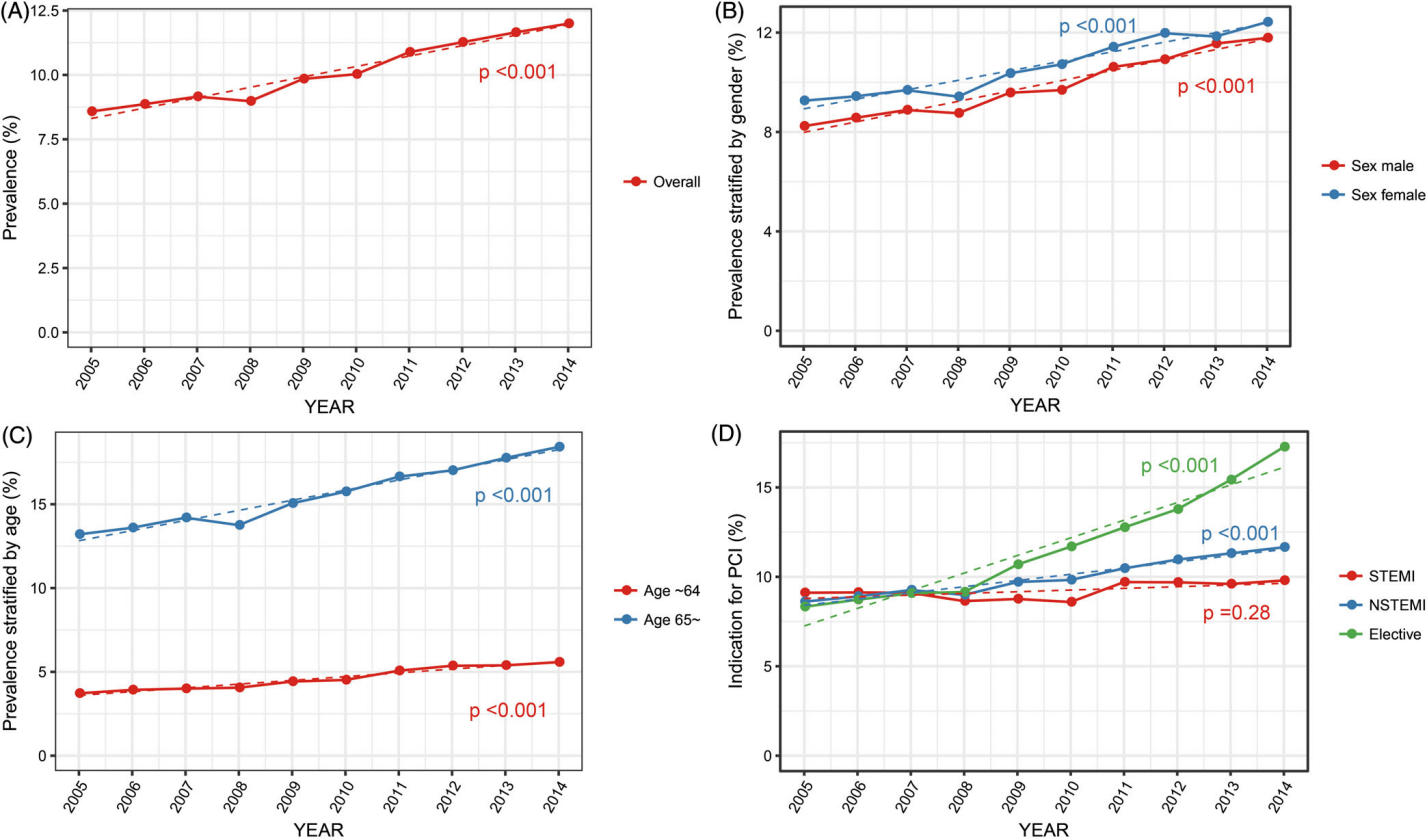
Trends in the prevalence of atrial fibrillation in hospitalizations undergoing percutaneous coronary intervention from 2005 to 2014. The prevalence of AF in A, the overall population that underwent PCI, as well as the stratified proportions according to, B, sex, C, age, and, D, indications for PCI. STEMI, ST‐segment elevation myocardial infarction; NSTEMI, non‐ST‐elevation myocardial infarction; AF, atrial fibrillation; PCI, percutaneous coronary intervention

Hospitalizations data from 6 272 232 weighted in‐patient PCI cases were included. Of these hospitalizations, 619 956 (9.9%) had AF and 5 652 276 (90.1%) did not have AF. A total of 33.7% of hospitalizations were women, and the median age of all hospitalizations was 65 years (56‐74 years). The prevalence of AF among hospitalizations who underwent PCI significantly increased from 8.6% in 2005 to 12.0% in 2014 (40.0% increase) (*P* < .001). Moreover, AF prevalence of all groups stratified by age, sex, and indication for PCI increased over time (*P* < .001 for all), except for those with ST‐segment elevation myocardial infarction (STEMI) (*P* = .28). By stratifying for age, AF prevalence among hospitalizations ≥65 years old markedly increased from 13.2% to 18.4% (39% increase), whereas this prevalence increased from 3.7% to 5.6% (51% increase) among those <65 years old during the study period. AF prevalence stratified by indications among STEMI hospitalizations remained very consistent, having only rose from 9.1% to 9.8% (7.7% increase), whereas the prevalence in NSTEMI hospitalizations increased from 8.6% to 11.7% (36.0% increase). A prominent increase in AF prevalence was observed in hospitalizations who underwent elective PCI (8.3% to 17.2%, 107% increase; Figure 1). Figure S2 shows that hospitalizations who underwent elective PCI had significant change toward a further increase in AF prevalence around 2008 by Davies test (*P* = .025). Figure S3 shows that the overall number of PCIs, PCIs in non‐AF hospitalizations, and PCIs in AF hospitalizations decreased by 42.0%, 44.2%, and 19.0%, respectively, with a significant downward trend (*P* < .001, *P* < .001, and *P* = .004, respectively).

Differences in patient demographics, hospital characteristics, and in‐hospital outcomes between AF and non‐AF hospitalizations are summarized in Table 1. At baseline, compared with non‐AF hospitalizations, AF hospitalizations were significantly more likely to have the following characteristics: older age, female, white, underwent elective PCIs, high household income, and have Medicare insurance. AF hospitalizations were also more likely to present with a prior history of having undergone coronary artery bypass grafting and have hypertension, congestive heart failure, peripheral artery disease, chronic renal failure, anemia, and dementia. Non‐AF hospitalizations were more likely to present with prior myocardial infarction/PCI, smoking history, obesity, dyslipidemia, and depression. Patient/hospital characteristics for propensity matching are summarized in Table 1. These characteristics became well‐balanced between the two matched groups (with and without AF, Figure S1), with the standardized difference for all variables being <3.0%. The trend stratified by AF for mean age and patient comorbidities in the weighted cohorts are summarized in Figure S4. There was a significant increase in the rates of congestive heart failure, diabetes, peripheral artery disease, chronic pulmonary disease, chronic kidney disease, prior myocardial infarction, and anemia; however, there was no significant difference in mean age among AF hospitalizations during the study period (Figure S4).

The in‐hospital procedures and outcomes for AF and non‐AF hospitalizations among the crude and propensity matched cohorts are presented in Table 2. AF hospitalizations undergoing PCI were more likely to undergo intra‐aortic balloon pump therapy and less likely to receive drug‐eluting stents in the crude and propensity‐matched cohorts (*P* < .001 for both). The rate of drug‐eluting stent use in AF hospitalizations who underwent PCI did not change during the study duration (*P* = .72; Figure S4).

**Table 2:** In‐hospital procedures and outcomes in hospitalizations with and without atrial fibrillation undergoing percutaneous coronary intervention

	Crude			Propensity‐matched		
	No AF	AF	*P* value	No AF	AF	*P* value
In‐hospital procedures						
DES	74.1	63.6	<.001	71.4	63.6	<.001
Bare metal stent	19.1	27.1	<.001	21.2	27.1	<.001
IABP	2.9	5.1	<.001	4.2	5.1	<.001
Multivessel disease	32.9	33	.86	33.8	33	<.001
Fractional flow reserve	0.8	1	<.001	0.8	1	<.001
Intravascular ultrasound	5.3	5.4	.28	5.1	5.4	.002
In‐hospital outcomes						
TIA/Stroke	0.8	1.6	<.001	1.2	1.6	<.001
Gastrointestinal bleeding	0.8	1.5	<.001	1.3	1.5	<001
Vascular complication	1	1.6	<.001	1.3	1.6	<.001
Blood transfusion	2.5	5.7	<.001	4.4	5.7	<.001
Cardiogenic shock	2.6	5.3	<.001	4.3	5.3	<.001
Acute kidney injury	5.1	10.4	<.001	9.6	10.4	<.001
Deep venous thrombosis	0.1	0.2	<.001	0.1	0.2	.003
Pulmonary embolism	0.2	0.3	<.001	0.3	0.3	.71
In‐hospital death	1.5	3.2	<.001	2.8	3.2	<.001
LOS, days	2 (1–3)	3 (2–6)	<.001	2 (1–5)	3 (2–6)	<.001
Non‐home discharge	10.3	25.5	<.001	19.8	25.5	<.001
Cost, $	19 320 (14395‐26 775)	22 425 (16058‐33 537)	<.001	20 590 (15047‐29 679)	22 425 (16058–33 537)	<.001

Abbreviations: AF, atrial fibrillation; CI, confidence interval; DES, drug‐eluting stent; IABP, intra‐aortic balloon pump; LOS, length‐of‐stay; OR, odds ratio; PCI, percutaneous coronary intervention; TIA, transient ischemic attack.

**Figure 2:**
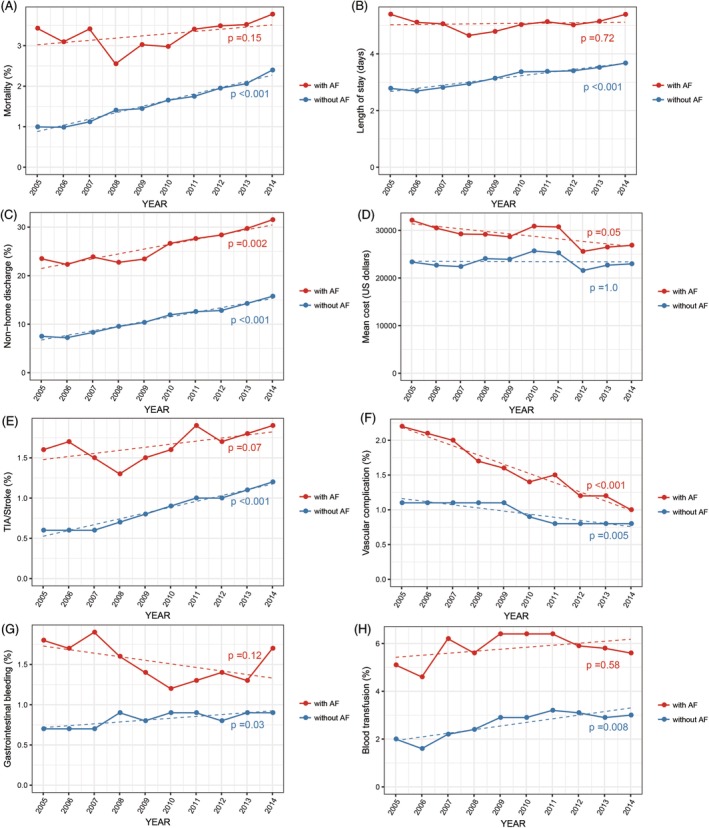
Trends in in‐hospital mortality and morbidity, as well as resource use and cost, among hospitalizations undergoing percutaneous coronary intervention stratified by atrial fibrillation from 2005 to 2014. Temporal trends in, A, in‐hospital mortality, B, length of stay, C, non‐home discharge, D, total cost, E, transient ischemic attack/stroke, F, vascular complications, G, gastrointestinal bleeding, and, H, blood transfusion in AF and non‐AF patients who underwent PCI. AF, atrial fibrillation; PCI, percutaneous coronary intervention

The results of multivariate logistic regression analysis for in‐hospital outcomes are shown in Table 3. In‐hospital mortality was significantly higher in AF hospitalizations who underwent PCI than in non‐AF hospitalizations (3.2% vs 1.5% and 3.2% vs 2.8%, respectively; adjusted odds ratio: 1.16; 95% confidence interval: 1.10‐1.21). There was no significant difference in the annual in‐hospital mortality in AF hospitalizations during the study period (*P* = .15; Figure 2). AF hospitalizations were more likely to have prolonged length of stay, non‐home discharge, higher hospitalization costs, TIA/stroke, gastrointestinal bleeding, vascular complications, blood transfusion, and cardiogenic shock (*P* < .001 for all). During the study period, there was a significant downward trend in vascular complications and an upward trend in non‐home discharge rates in AF hospitalizations (*P* < .001 and *P* = .002, respectively). However, there were no significant changes in the trends of length of stay, medical costs, TIA/stroke, gastrointestinal bleeding, and blood transfusion in AF hospitalizations during the study period. Increased rates of in‐hospital mortality, in‐hospital morbidity, costs, and prolonged length of stay in AF hospitalizations were consistently observed throughout the study period (Figure 2).

**Table 3:** Multivariate logistic regression analysis for in‐hospital outcomes among hospitalizations either with or without AF undergoing PCI using a propensity‐matched cohort

Characteristic	OR	95% CI	*P* value
TIA/stroke	1.42	1.33‐1.52	<.001
Gastrointestinal bleeding	1.14	1.07‐1.22	<.001
Vascular complication	1.31	1.22‐1.40	<.001
Blood transfusion	1.37	1.32‐1.42	<.001
Cardiogenic shock	1.28	1.23‐1.33	<.001
Acute kidney injury	1.11	1.08‐1.14	<.001
Deep venous thrombosis	1.32	1.09‐1.59	.005
Pulmonary embolism	1.01	0.88‐1.18	.84
In‐hospital death	1.16	1.10‐1.21	<.001
Non‐home discharge	1.49	1.46‐1.52	<.001

Abbreviations: AF, atrial fibrillation; CI, confidence interval; OR, odds ratio; PCI, percutaneous coronary intervention; TIA, transient ischemic attack.

## 4 DISCUSSION

From the estimated 6 272 232 hospitalizations who underwent PCI from 2005 to 2014, we found several important results by conducting a detailed AF‐focused analysis: (a) AF prevalence in patients who underwent PCI steadily increased from 8.6% in 2005 to 12.0% in 2014, with a marked increase among elderly patients and those who underwent elective PCI; (b) in‐hospital mortality and morbidity, such as TIA/stroke, vascular complications, and cardiogenic shock, were independently associated with AF even after adjustment for patient/hospital covariates; (c) the in‐hospital mortality, non‐home discharge, length of stay, and in‐hospital costs were higher in AF patients than in non‐AF patients; (d) the vascular complications in AF patients had decreased during the study period. However, in‐hospital mortality, stroke rate, length of stay, and medical costs in AF patients remained consistent over time, and the rate of non‐home discharge significantly increased. To the best of our knowledge, this study is the largest and the most recent evaluation of trends and outcomes among AF patients who have undergone PCI.

In this nationwide study, almost 1 in 10 patients had AF during their hospitalization, and AF prevalence steadily increased by 14.0% over the study period. The reason for the increase in AF among patients who underwent PCI might be that these individuals were more likely to have clinically predisposed AF comorbidities^32^ such as heart failure, hypertension, diabetes, chronic pulmonary disease, and a history of myocardial infarction. In addition, the age‐stratified analysis showed that a higher AF prevalence was observed in patients ≥65 years old, despite a consistent mean age of AF patients. This suggests that a greater accumulation of comorbidities in the elderly largely contributes to the overall increase in AF prevalence in this population. In addition, prevalence of AF has increased among the general population of other developed countries, possibly due to similar reasons,^33^, ^34^ indicating that AF in patients undergoing PCI will also increase.

When stratifying by PCI indications, AF prevalence in STEMI patients remained nearly the same (only increasing from 9.1% to 9.8% between 2005 and 2014). This finding is consistent with a previous multinational study of acute coronary syndrome, which reported a slightly decreased prevalence from 12.9% in 2000 to 11.1% in 2007,^3^ as well as with a US community‐based study of acute myocardial infarction that reported an almost invariable AF prevalence from 1975 to 2005.^35^ However, we observed a significant increase in AF prevalence in non‐ST‐segment elevation myocardial infarction (NSTEMI) and elective PCI patients from 8.6% to 11.7% and 8.3% to 17.2%, respectively. Furthermore, a significant accelerated increase in AF prevalence around 2008 was detected among patients undergoing elective PCI. These findings might be secondary to the introduction of appropriateness criteria to optimize the benefit‐risk balance for PCI,^36^ the shift toward more out‐patient care for PCI,^28^ and the resulting decrease in in‐patient PCIs, particularly for non‐acute indications in the U.S. However, further studies are needed to systemically and comprehensively clarify the disparities and risk profiles in temporal changes in AF patients who underwent PCI in terms of clinical presentation types at the time of PCI. Collectively, our data clearly show the substantial increase of AF prevalence and emphasizes the need for improving quality of care by examining the largest well‐validated dataset in the contemporary era.

Our data identified racial disparities in the prevalence of AF in patients undergoing PCI; a higher prevalence of AF was observed in white than in black and Hispanic patients. Previous studies also reported that AF is less prevalent and left atrial appendage occlusions are performed less frequently in black and Hispanic than in white patients.^37^, ^38^, ^39^ Predisposing genetic factors might explain these racial disparities in prevalence of AF.^40^ However, further studies are needed to clarify the issues.

Our study reported a 1.5% in‐hospital mortality among non‐AF patients, compared to a 3.2% risk among AF patients in the crude cohort. These rates were comparable with a previous study that analyzed a prospective multicenter registry in Michigan, which assessed AF history among patients who underwent PCI and reported in‐hospital mortality of 1.3% and 3.2% (non‐AF vs AF patients).^2^ Consistent with previous studies that assessed the impact of AF on short‐term mortality,^2^, ^4^, ^29^, ^30^ our large nationwide propensity‐matched analysis and ad‐hoc multivariate analysis confirmed that AF independently influenced in‐hospital mortality, with this excess in mortality being consistently observed over the study period.

Prior studies have reported that both pre‐existing AF and new‐onset AF are associated with an increased risk of significant in‐hospital mortality as well as cardiovascular/bleeding complications in the setting of PCI and acute coronary syndrome.^2^, ^3^, ^5^, ^30^ In addition, it has been reported that ~90% of new‐onset AF cases occur within 4 days of admission of STEMI, which underscores the fact that new‐onset AF generally occurs immediately after the coronary event.^5^ It has been theorized that AF is a clinical consequence of atrial fibrosis caused by various predisposing factors such as valvular insufficiency, diastolic dysfunction, and hypertension, and that AF itself promotes the atrial fibrosis.^41^ It should be noted that several AF associated issues, including echocardiography findings and periprocedural treatments, were not adjusted in this study. Higher in‐hospital mortality might be due to the association between AF and thrombotic events or poorer hemodynamic status, as well as underlying pathophysiology.

In this study, we showed that AF patients were at a higher risk for thrombotic and bleeding complications, such as TIA/stroke, gastrointestinal bleeding, blood transfusion, and vascular complications in the crude cohort, which is consistent with results of prior studies.^2^, ^4^, ^31^ The association between AF and these in‐hospital complications remained significant after vigorous propensity matching between AF and non‐AF patients; these results were similar to those of previous studies.^5^, ^32^ Sutton et al. also reported a higher rate of stroke (0.5% vs 0.4%, *P* = .345), bleeding event (3.7% vs 2.8%, *P* < .001), blood transfusion (5.2% vs 4.6%, *P* = .025), and vascular complications (0.7% vs 0.5%, *P* = .064) in AF patients using propensity‐matched cohorts.^2^

Higher rates of thrombotic and bleeding complications might be related to the fact that AF itself is associated with an increased risk of thromboembolisms, and AF patients are more likely to take oral anticoagulants plus dual antiplatelet drugs. Previous guidelines during the study period recommended “triple therapy” for AF patients who underwent PCI and those who had a high CHADS2 score.^6^ In clinical settings, clinicians tailored the periprocedural antithrombotic therapy to mitigate the risk of bleeding complications. Antiplatelet drugs and anticoagulants were often discontinued when bleeding complications presented or were likely to present. In 2009, Lopes et al. reported that only 43% of patients undergo triple therapy at discharge without discontinuing antiplatelet drugs. They also reported that patients with higher CHADS2 scores were more likely to paradoxically discontinue the anticoagulants at discharge, possibly due to concerns for bleeding risks.^5^ In addition, our propensity‐matched analysis showed that AF patients are less likely to receive drug‐eluting stents, which is congruent with the results of a previous study,^2^ probably due to similar concerns with an increased risk of bleeding.

Consistent with the rapid evolution of the antithrombotic strategy for AF and PCI, guidelines and consensus documents incorporating new evidences are frequently updated.^37^ More recent guidelines recommend considering a short duration of triple therapy or even avoiding the therapy altogether based on patients' thromboembolic and bleeding risk.^33^, ^42^ Several key randomized trials support the elevated safety of direct oral anticoagulant intake with single antiplatelet therapy (double antithrombotic therapy) compared with a regimen of triple therapy with vitamin K agonists.^7^, ^12^, ^13^ Furthermore, during the study period, despite the significantly increased rates of TIA/stroke and thrombotic complications, possibly caused by higher comorbidity effects in AF patients undergoing PCI, the rate of vascular complication significantly decreased, and the rates of gastrointestinal bleeding/blood transfusion remained consistent. This encouraging result might be partially due to triple therapy being utilized less, and further studies are warranted to investigate how current guidelines and the use of direct oral coagulants affect in‐hospital bleeding complications. Additionally, in the propensity‐matched model, we showed that AF patients used more resources; AF patients showed prolonged length of stay, higher in‐hospital costs, and higher rates of non‐home discharge than non‐AF patients. Collectively, our study underscored that AF patients who underwent PCI were susceptible to both thrombotic and bleeding events, even during hospitalization, and that there is a continued need for improving treatment strategies and reducing costs for these patients.

### 4.1 Study limitations

Our study has a number of limitations, which are similar to those of other studies using large administrative databases. First, this study has the potential for any bias inherent to retrospective observational studies. Second, there may be substantial coding errors and coding bias derived from the ICD‐9‐CM codes. However, similar approaches for capturing data on the prevalence AF and PCI were used in previous studies.^19^, ^20^, ^22^, ^23^ In addition, several outcome measures examined in our study, including in‐hospital mortality, length of stay, cost, and non‐home discharge, are rarely miscoded. Third, the NIS does not record data regarding diagnostic findings of coronary angiography, procedural characteristics of PCI, peri‐procedural medications, electrocardiograms, left ventricular ejection fraction by echocardiography, or laboratory variables. In addition, data are not available in the NIS to calculate the CHADS2 scores for assessing the risk of thromboembolism. Fourth, the propensity‐matching and ad‐hoc multivariate analysis did not account for antithrombotic regimen and procedural characteristics. However, previous study used a similar approach.^2^ Finally, we could not distinguish between preexisting AF and new‐onset AF. However, it has been reported that both preexisting AF and new‐onset AF are associated with worse clinical outcomes^2^, ^3^, ^5^ and that previously undetected preexisting AF may not preclude a case of paroxysmal AF. Despite these limitations, we believe that our study was strengthened by using the NIS to analyze real‐world clinical practice with a nation‐wide estimate that represents in‐patient data in the U.S.

## 5 CONCLUSIONS

Using the largest nationally representative cohort of trends in demographics and outcomes for AF patients who underwent PCI, we demonstrated that the overall AF prevalence among patients who underwent PCI continuously increased, particularly in elderly patients and those who underwent elective PCIs. AF patients continued to show high in‐hospital mortality, morbidity, and medical costs over the recent decade. This study highlights the continued need to identify preventive and management strategies to reduce risks and costs associated with AF patients undergoing PCI.

## CONFLICT OF INTEREST

The authors declare no potential conflict of interests.

## Supporting information


**Appendix** S1. Supporting Information
