# Supplementary material for: Temporal trends in prevalence and outcomes of atrial fibrillation in patients undergoing percutaneous coronary intervention
Source: Clin Cardiol. 2019 Nov 6;43(1):33–42. doi: 10.1002/clc.23285 (PMC6954373; doi:10.1002/clc.23285)
Supplement: Supplementary file 1 — Appendix S1. Supporting Information [file CLC-43-33-s001.docx]

**SUPPLEMENTAL MATERIALS**

**Temporal trends in prevalence and outcomes of atrial fibrillation in patients undergoing percutaneous coronary intervention**

Yusuke Morita^1*^, Toka Hamaguchi^1^, Tetsuya Haruna^1^, Yoshisumi Haruna^1^, Eisaku Nakane^1^, Yuhei Yamaji^1^, Hideyuki Hayashi^1^, Michiya Hanyu^1^, Moriaki Inoko^1^

Cardiovascular Center, Tazuke Kofukai Foundation, Medical Research Institute, Kitano Hospital

**^*^Corresponding author:** Yusuke Morita, MD, PhD

Cardiovascular Center, Tazuke Kofukai Foundation, Medical Research Institute, Kitano Hospital, 2-4-20 Ohgimachi, Kita-ku, Osaka, JAPAN, 530-8480. Tel: +81-6-6312-1221, Fax: +81-6-6312-8867. E-mail: [ysk-morita@kitano-hp.or.jp](mailto:ysk-morita@kitano-hp.or.jp)

**Running title:** Atrial fibrillation in percutaneous coronary intervention

**Supplemental Table**

**Table S1**. International Classification of Diseases, Ninth Edition, Clinical Modification, Elixhauser Comorbidity Software, and Clinical Classification Software were used to identify patient/hospital characteristics

| Characteristics | Source | Codes |
| --- | --- | --- |
| Indication for PCI |  |  |
| STEMI | ICD-9-CM | 41000, 41001, 41010, 41011, 41020, 41021, 41030, 41031, 41040, 41041, 41050, 41051, 41060, 41061, 41080, 41081, 41090, 41091 |
| NSTEMI/UAP | ICD-9-CM | 41070, 41071, 4111 |
| Comorbidities |  |  |
| Family history of coronary artery disease | ICD-9-CM | V173 |
| Prior MI | ICD-9-CM | 412 |
| Prior PCI | ICD-9-CM | V4582 |
| Prior CABG | ICD-9-CM | V4581 |
| Carotid artery disease | ICD-9-CM | 43310 |
| Smoking history | ICD-9-CM | V1582, 3051 |
| Dyslipidemia | CCS Diagnoses | 53 |
| Dementia | ICD-9-CM | 2900, 29010-29013, 29020-29021, 2903, 29040-29043, 2908, 2909, 29410, 29411, 29420, 29421, 2948, 3310, 33111, 33119, 33182, 797 |
| In-hospital procedures |  |  |
| Baremetal stent | ICD-9-PCS | 3606 |
| DES | ICD-9-PCS | 3607 |
| IABP | ICD-9-PCS | 3761 |
| Multivessel PCI | ICD-9-PCS | 0041, 0042, 0043, 0046, 0047, 0048 |
| Fractional flow reserve | ICD-9-PCS | 0059 |
| Intravascular ultrasound | ICD-9-PCS | 0024 |
| In-hospital outcomes |  |  |
| TIA/stroke | ICD-9-CM | 9970, 99700, 99701, 99702, 430, 431, 432, 4320, 4321, 4329, 43301, 43311, 43321, 43331, 43381, 43391, 43401, 43411, 43491, 4350, 4351, 4352, 4353, 4358, 4359, 436 |
| Gastrointestinal bleeding | ICD-9-CM | 5780, 5781, 5789 |
| Vascular complications | ICD-9-CM | 9982, 9992, 99771, 99772, 99779, 4470, 86804 |
|  | ICD-9-PCS | 3931, 3941, 3949, 3952, 3953, 3956, 3957, 3958, 3959, 3979 |
| Blood transfusion | CCS Procedures | 222 |
| Cardiogenic shock | ICD-9-CM | 78551 |
| Acute kidney injury | ICD-9-CM | 5845, 5846, 5847, 5848, 5849 |
| Deep venous thrombosis | ICD-9-CM | 4511, 4512, 45181, 4519, 4531, 4532, 4538, 4539 |
| Pulmonary embolism | ICD-9-CM | 41511, 41512, 41513, 41519 |

CABG, Coronary artery bypass grafting; CAD, Coronary artery disease; CCS, Clinical Classification Software;

DES, Drug-eluting stent; IABP, Intra-aortic balloon pumping; ICD9-CM, International Classification of Diseases, Ninth Edition, Clinical Modification; MI, Myocardial infarction; NSTEMI, Non ST-elevation myocardial infarction; PCI, percutaneous coronary intervention; STEMI, ST-elevation myocardial infarction;

TIA, Transient ischemic attack; UAP, Unstable angina

**Supplemental Figures and Figure Legends**


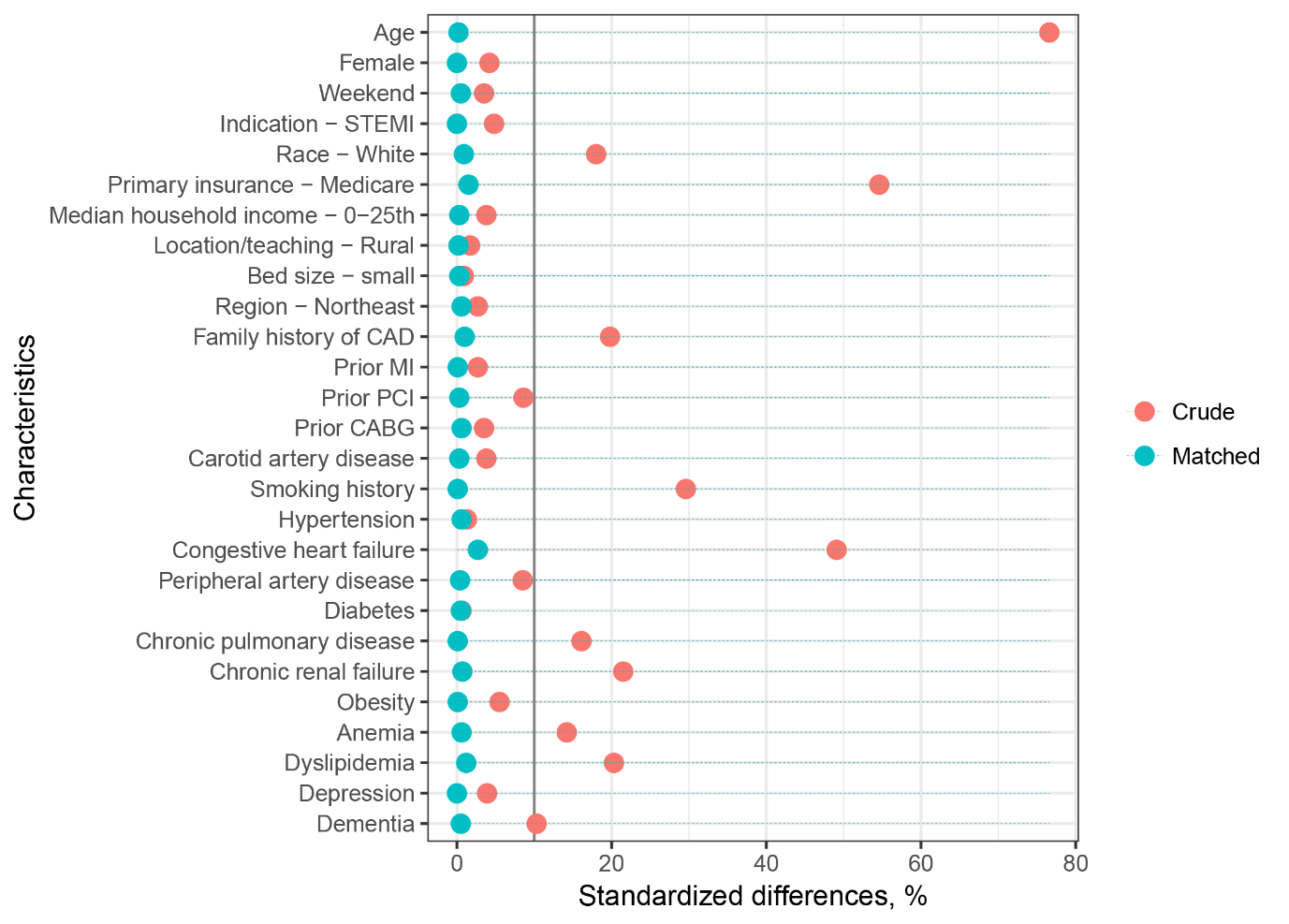


**Figure S1.** Dot plot presenting the standardised differences of baseline patient/hospital characteristics among crude and propensity score-matched cohorts.


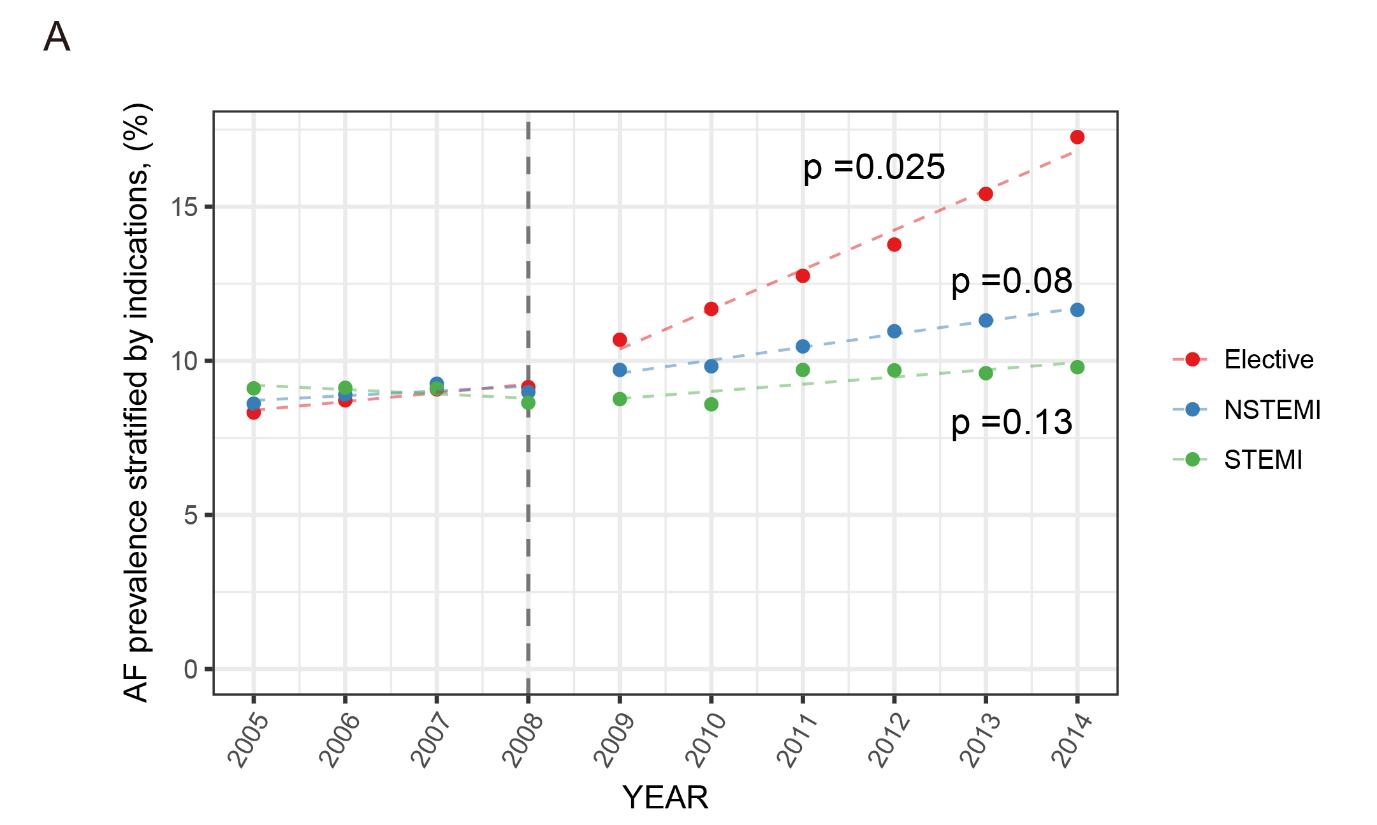


**Figure S2.** Trends in the prevalence of atrial fibrillation (AF) in patients undergoing percutaneous coronary intervention (PCI) stratified by the indications during the study period 2005 to 2008 and 2009 to 2014.

Significant difference in the slope around 2008 among elective PCIs was detected by Davies test (p=0.025), but no significant differences were detectable among non-ST-elevation myocardial infarction (NSTEMI) and ST-elevation myocardial infarction (STEMI) group (p=0.08 and p=0.13, respectively).


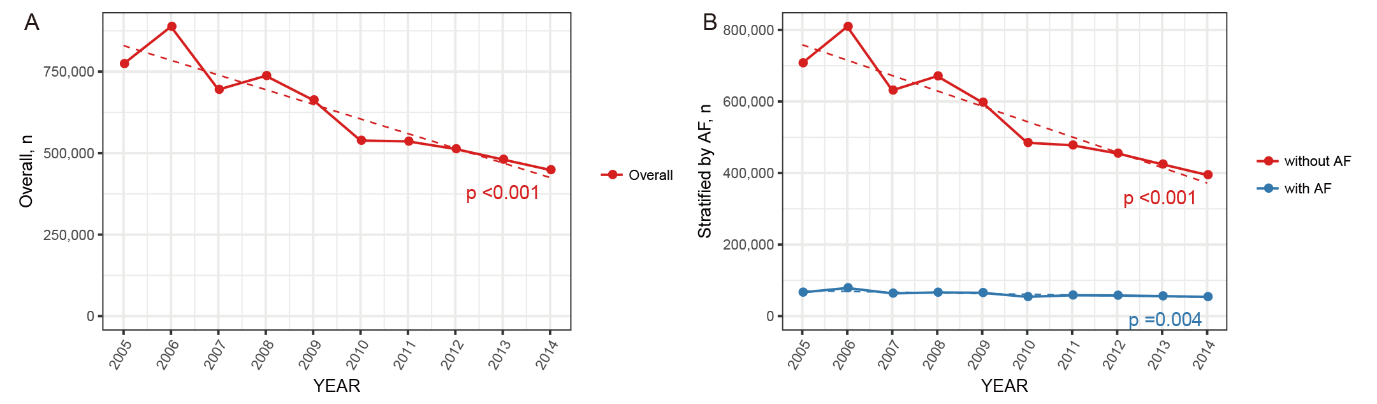


**Figure S3.** Overall (A) and stratified by atrial fibrillation (AF) (B) percutaneous coronary intervention (PCI) volumes from 2005 to 2014.


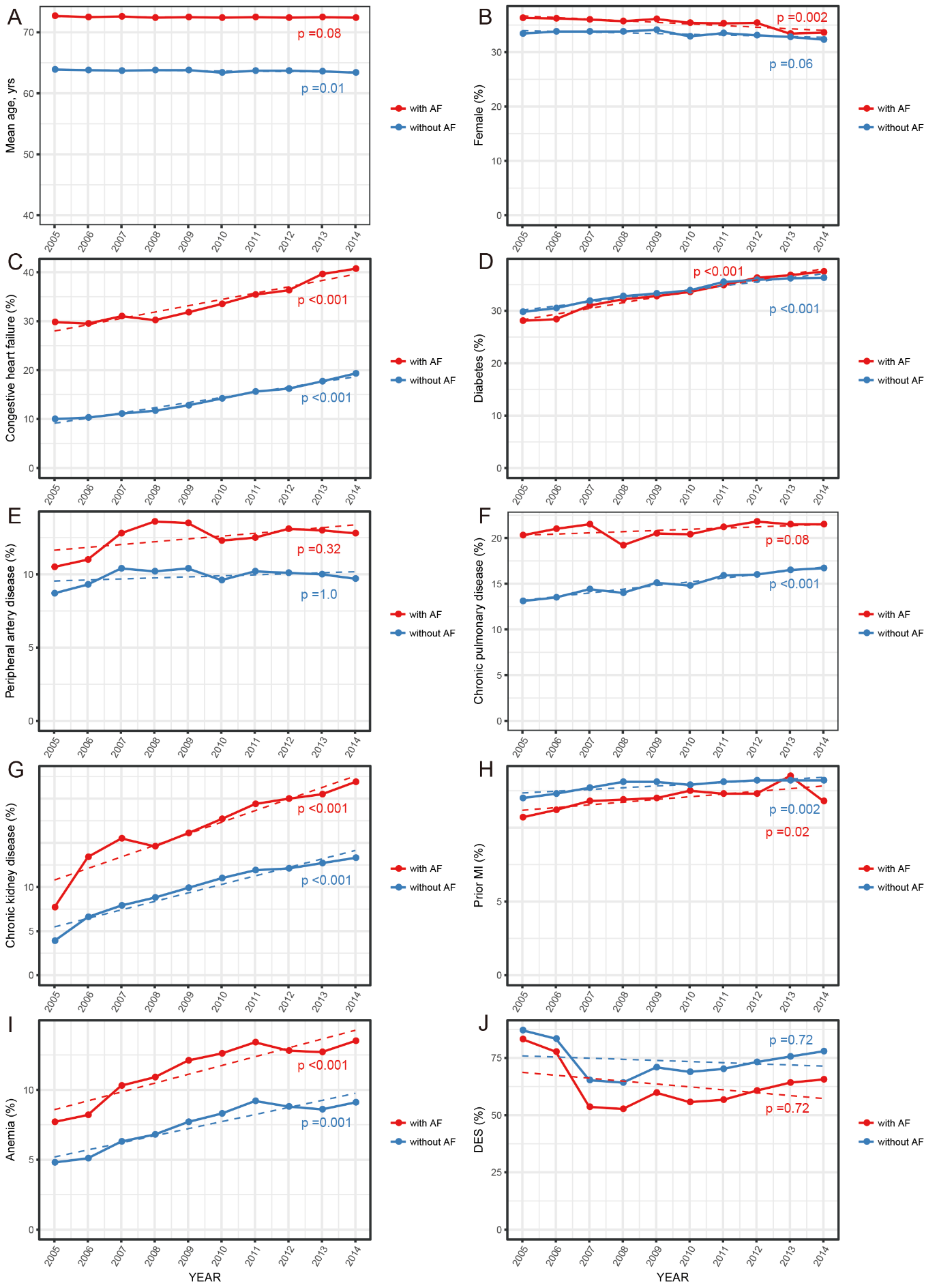


**Figure S4.** Trends of patient characteristics stratified by atrial fibrillation (AF)

(A) Mean age, rates of (B) female, (C) congestive heart failure, (D) diabetes, (E) peripheral artery disease, (F) chronic pulmonary disease, (G) chronic kidney disease, (H) prior myocardial infarction (MI), (I) anemia, and (I) usage of drug-eluting stent (DES).
